# Impact of Introducing a Multiplex Polymerase Chain Reaction Blood Culture Panel on Anti-Methicillin-Resistant Staphylococcus aureus (MRSA) and Carbapenem Antimicrobial Agents in a Children’s Hospital

**DOI:** 10.7759/cureus.66282

**Published:** 2024-08-06

**Authors:** Kenta Kuruma, Hanako Funakoshi, Meiwa Shibata, Keiko Okita, Junichi Suwa, Tomoyuki Tame, Yuho Horikoshi

**Affiliations:** 1 Division of Infectious Diseases, Tokyo Metropolitan Children’s Medical Center, Tokyo, JPN; 2 Department of Infectious Diseases and General Pediatrics, Nagano Children’s Hospital, Nagano, JPN; 3 Division of Pharmacy, Tokyo Metropolitan Children’s Medical Center, Tokyo, JPN; 4 Division of Laboratory, Tokyo Metropolitan Children’s Medical Center, Tokyo, JPN

**Keywords:** antibiotics, antimicrobial stewardship program, bacteremia, blood culture, multiplex pcr

## Abstract

Background: With the advent of multiplex polymerase chain reaction (PCR) using samples from a positive blood culture, the time required to identify a pathogen has significantly shortened to a few hours. It can help us select appropriate antimicrobial agents more quickly. The present study aimed to assess the impact of using a multiplex PCR blood culture panel on the appropriate administration of antimicrobial agents.

Methods: Patients aged <16 years with culture-confirmed bacteremia at Tokyo Metropolitan Children's Medical Center were enrolled. A pre-intervention period (period I: December 2016 to December 2018) and a post-intervention period with multiplex PCR use for the confirmation of positive blood cultures (period II: December 2019 to December 2021) were compared for their effect on the use of antimicrobial agents for gram-positive cocci (GPC) and gram-negative rod (GNR) bacteremia. Data on patient background, blood culture results, and antimicrobial use were retrospectively collected from electronic medical records.

Results: Periods I and II had 174 and 154 patients, respectively. The median age at periods I and II was 14 (IQR 2-82) months and 12 (IQR 1-78) months, respectively. GPC bacteremia during periods I and II occurred in 140 and 115 patients, respectively. GNR during periods I and II occurred in 34 and 39 patients, respectively. Neither the vancomycin-resistance genes A/B nor the carbapenem-resistance gene were detected. The use of antimicrobial agents against anti-methicillin-resistant *Staphylococcus aureus* (MRSA) for GPC bacteremia decreased from 103/140 cases (73%) in period I to 56/115 cases (49%) in period II (p=0.047). The use of carbapenems for GNR bacteremia did not change significantly, at 23/34 (68%) in period I and 34/39 (87%) in period II (p=0.47).

Conclusion: Introducing multiplex PCR for pediatric bacteremia decreased the use of anti-MRSA antimicrobial agents but not of carbapenems.

## Introduction

The increase in infections caused by antimicrobial-resistant bacteria has become a worldwide problem. In 2015, infections caused by antibiotic-resistant bacteria were estimated to result in a significant number of deaths across Europe, particularly affecting infants and the elderly [1-3]. The appropriate use of antimicrobial agents is a critical countermeasure to the emergence of resistant microorganisms [4,5], and the importance of antimicrobial stewardship is increasingly being recognized. Bacterial identification and susceptibility testing using the conventional blood culture method with biochemical identification typically require two to three days when an automated machine is used in an in-house laboratory. Sepsis is a life-threatening condition that requires a rapid response. Guidelines recommend starting antimicrobial agents within three hours of recognizing sepsis [6,7]. Initial treatment often uses broad-spectrum antimicrobials until bacteria are identified. However, the use of broad-spectrum antimicrobials should be minimized to prevent resistant strains [8].

With the advent of mass spectrometry and multiplex polymerase chain reaction (PCR) using samples from a positive blood culture, the time required to identify a pathogen has significantly shortened to a few hours. Recently, mass spectrometers have been used to quickly identify bacteria from positive blood cultures, but they cannot detect resistance genes, leading to inappropriate antimicrobial use until susceptibility is determined. Although culture-based techniques remain the gold standard, multiplex PCR blood panels are increasingly used as a complement. Therefore, more facilities are adopting multiplex PCR. Detecting resistance genes with multiplex PCR can also rapidly yield susceptibility results [9]. These advances in microbiological techniques can enhance diagnostic antimicrobial stewardship to enable optimal antimicrobial selection during the early stages of treatment [10]. However, reports of the impact of diagnostic antimicrobial stewardship in pediatrics are still limited [11]. The present study aimed to determine the clinical impact of the blood culture multiplex PCR panel as a diagnostic antimicrobial stewardship tool on antimicrobial use in pediatric patients with bacteremia by comparing it with conventional identification methods and susceptibility testing. We investigated whether anti-methicillin-resistant *Staphylococcus aureus* (MRSA) antimicrobial agents were used when gram-positive cocci (GPC) was detected in a positive blood culture and whether carbapenems were used when gram-negative rod (GNR) was detected. In this study, appropriate antimicrobial use was defined as the use of anti-MRSA antimicrobial agents and carbapenems specifically for bacteria identified in blood cultures that require these antimicrobials.

## Materials and methods

Study protocol

In the pre-intervention period (period I), samples of blood cultures determined to be positive for the target pathogen by an automated incubating system, BACT/ALERT® 3D (BioMerieux Japan, Tokyo, Japan), were gram-stained for confirmation by a laboratory technician. Based on a current laboratory manual, GPC bacteremia and GNR bacteremia were empirically treated with vancomycin and meropenem, pending antimicrobial susceptibility results. The results of identification and susceptibility testing for pathogens normally returned within two to three days, after which the antimicrobial agents were switched to a definitive therapy or discontinued if contamination was suspected. In the post-intervention period (period II), a multiplex PCR panel for a positive blood culture was used for the rapid identification of common bacteria and fungi with resistance genes within an hour after the positivity of a blood culture was confirmed. The present study compared the choice of antimicrobial agent for pediatric bacteremia between a pre- and post-intervention period. The Institutional Review Board of Tokyo Metropolitan Children’s Medical Center approved this study (approval number: 2022b-41).

Subjects

Patients aged 15 years or less at the study center with bacteremia and a positive blood culture result for GPC or GNR were included in periods I (December 2016 to December 2018) and II (December 2019 to December 20). In patients with multiple positive blood cultures in the same episode of bacteremia, the first positive culture was included. Cases of bacteremia caused by gram-positive bacilli or gram-negative cocci were not included. If multiple bacteria were detected, they were also excluded.

Data on the patient’s demographic background, antimicrobial use, and 30-day mortality rate were collected from electronic medical charts retrospectively. The primary outcome was the use of anti-MRSA antimicrobial agents for GPC bacteremia and the use of carbapenems for GNR bacteremia in periods I and II. The secondary outcome was the use of anti-MRSA antimicrobial agents and carbapenems in periods I and II, when these drugs were appropriate treatments for GPC and GNR bacteremia, respectively. The target cases were those of methicillin-resistant GPC bacteremia and carbapenem-susceptible GNR bacteremia with resistance to other beta-lactams (e.g., extended-spectrum beta-lactamase (ESBL)-producing GNR). Additionally, we investigated GNRs in relation to the days of therapy (DOT) for carbapenems. DOT was defined as the total number of days carbapenems were used divided by the number of study subjects.

Multiplex PCR blood culture panel

Automated multiplex PCR for positive blood cultures was done with the FilmArrayⓇ Torch system (BioMerieux Japan, Tokyo, Japan) using a BioFireⓇ blood culture panel (BioFire Diagnostics, Salt Lake City, USA). Culture fluid from a positive blood culture bottle was extracted and amplified with multiplex PCR. Table 1 shows a list of microorganisms and genes that can be detected by FilmArrayⓇ.

**Table 1:** Microorganisms and genes detected by FilmArray® Twenty-four commonly targeted pathogens: gram-positive bacteria (n=8), gram-negative bacteria (n=11), *Candida *species (n=5), and pathogens with antimicrobial resistance genes (n=3)

Gram-positive cocci	Gram-negative cocci	Fungus
Staphylococcus spp.	Enterobacteriaceae	Candida albicans
S. aureus	E. coli	Candida glabrata
*Enterococcus *spp.	K. pneumoniae	Candida krusei
Listeria monocytogenes	K. oxytoca	Candida parapsilosis
*Streptococcus *spp.	Enterobacter cloacae	Candida tropicalis
S. pneumoniae	S. marcescens	Antimicrobial resistance genes
	Proteus	
S. pyogenes	Neisseria meningitidis	mecA
S. agalactiae	P. aeruginosa	vanA
	Haemophilus influenzae	vanB
	Acinetobacter baumannii	KPC

Statistical analyses

All statistical analyses were conducted using SPSS Statistics version 22 (IBM SPSS Statistics, Version 22 for Windows; IBM Corp., Armonk, N.Y., USA). Parameters were compared using the χ square test or Fisher's exact test as needed. P<0.05 was considered to indicate statistical significance. Data in the tables are mainly expressed as the median and the first and third quartile values.

## Results

Subjects' profile

Patients in periods I and II numbered 174 and 154, respectively. The baseline characteristics of the patients did not differ significantly between the periods (Tables 2-3). There was also no significant difference in 30-day mortality between the periods.

**Table 2:** Subject profile No statistically significant difference in age, sex, or underlying disease was found. GPC: gram-positive cocci, GNR: gram-negative rod

	Period Ⅰ (N=174)	Period Ⅱ (N=154)	P-value
Age (month) (IQR)	14 (2-82)	12 (1-78)	-
% females	76 (44%)	64 (42%)	0.70
GPC	140 (81%)	115 (75%)	0.70
GNR	34 (19%)	39 (25%)	0.21

**Table 3:** Underlying diseases in patients with GPC patients and GNR-positive patients No statistically significant difference in underlying disease was found. PICU: pediatric intensive care unit, NICU: neonatal intensive care unit, GPC: gram-positive cocci, GNR: gram-negative rod

GPC	Period Ⅰ (N=140)	Period Ⅱ (N=115)	P-value
Congenital heart disease	21 (15%)	12 (10%)	0.14
Neutropenia (<500/μL)	11 (8%)	8 (7%)	0.59
PICU	18 (13%)	16 (14%)	0.91
NICU	21 (15%)	26 (22%)	0.24
Central venous catheter	53 (38%)	38 (33%)	0.084

GNR	Period Ⅰ (N=34)	Period Ⅱ (N=39)	P-value
Congenital heart disease	8 (24%)	5 (13%)	0.23
Neutropenia (<500/μL)	5 (15%)	9 (23%)	0.36
PICU	5 (15%)	9 (23%)	0.36
NICU	11 (32%)	11 (28%)	0.70
Central venous catheter	20 (59%)	26 (67%)	0.49

GPC

GPC bacteremia was reported in 140 cases during period I and 115 cases during period II (Table 4). Anti-MRSA antimicrobial agents were used in 103 of these cases in period I and 56 cases in period II. The use of anti-MRSA antimicrobial agents for GPC bacteremia significantly decreased from 73% in period Ⅰ to 49% in period Ⅱ (p=0.047). Either period, anti-MRSA antimicrobial agents were used for all patients with methicillin-resistant GPC bacteremia. All isolates of MRSA and *methicillin-resistant coagulase-negative staphylococci* (MRCNS) harbored the *mecA* gene, which was detected by multiplex PCR.

**Table 4:** GPC detected by multiplex PCR Number of bacteria species isolated per period MSSA: methicillin-sensitive *Staphylococcus aureus,* MRSA: methicillin-resistant *Staphylococcus aureus*, CNS: coagulase-negative *Staphylococcus aureus,* MRCNS: methicillin-resistant coagulase-negative *Staphylococcus aureus*, GAS: group A *Streptococcus*, PSSP: penicillin-sensitive *Streptococcus pneumoniae*, PRSP: penicillin-resistant *Streptococcus pneumoniae*, GBS: group B *Streptococcus, *GPC: gram-positive cocci, PCR: polymerase chain reaction

	Period Ⅰ	Period Ⅱ
Micrococcus sp.	2	1
MRSA	11	5
MSSA	35	17
MRCNS	45	47
CNS	23	14
GAS	2	2
PSSP	10	6
PRSP	3	1
*α-Streptococcus* sp.	7	11
GBS	2	8
Enterococcus faecalis	0	3

GNR

GNR bacteremia was reported in 34 cases during period I and 39 cases during period II (Table 5). Carbapenems were used in 23 of these cases (68%) in period I and 34 cases (84%) in period II. There was no statistically significant difference in the use of carbapenems for GNR bacteremia between periods Ⅰ and Ⅱ (p=0.47). DOT for carbapenems was 4.41 in period I and 5.05 in period II. There was no statistically significant difference between the two periods (p=0.98). Either period, carbapenems were used in all patients with ESBL-producing GNR bacteremia.

**Table 5:** GNR detected by multiplex PCR Number of ESBL-producing bacteria and bacteria species per period GNR: gram-negative rod, PCR: polymerase chain reaction, ESBL: extended-spectrum beta-lactamase

	Period Ⅰ	Period Ⅱ
Elizabethkingia meningoseptica	1	0
Enterobacter cloacae	2	2
Escherichia coli	16	14
Haemophilus influenzae	1	0
*Helicobacter *spp.	1	0
Klebsiella oxytoca	3	2
Klebsiella pneumoniae	2	4
Klebsiella aerogenes	2	1
Pseudomonas aeruginosa	2	6
Serratia marcescens	2	2
Sphingomonas paucimobilis	2	0
Achromobacter xylosoxidans	0	2
*Acinetobacter *spp*.*	0	1
Campylobacter jejuni	0	1
Morganella morganii	0	1
*Salmonella *spp*.*	0	3
Number of ESBL-producing bacteria	2	2

## Discussion

The advantage of multiplex PCR is its ability to rapidly identify staphylococci by detecting the *mecA* gene [11,12], which may contribute to reducing the use of anti-MRSA agents in two ways. In the present study, MRSA bacteremia comprised only 20.6% (12/58) of all cases of *S. aureus* bacteremia. The detection of *mecA* matched conventional susceptibility testing results. Thus, the first way in which multiplex PCR may reduce the need for anti-MRSA agents may be by rapidly differentiating between MSSA and MRSA, thereby also optimizing the use of anti-MRSA agents. Another way might be through the rapid identification of coagulase-negative staphylococci (CNS), which comprised 62.5% (107/171) of GPC bacteremia cases in the present study. The introduction of multiplex PCR allowed rapid identification of the CNS, making it easier to determine contamination. This is thought to have led to a reduction in the use of anti-MRSA agents. This might have had a large impact, as CNS was more frequently isolated (Table 4). Although previous studies of adults have yielded similar results [10], multiplex PCR in the pediatric setting, where contamination is more prevalent, is more likely to contribute to decreasing the use of anti-MRSA antimicrobial agents.

The present study did not find a reduction in the use of carbapenems for GNR bacteremia. A previous study reported a decrease in the use of anti-*Pseudomonas aeruginosa* agents and carbapenem antimicrobials in adults with GNR bacteremia as a result of the combined use of local bacteremia guidelines and multiplex PCR to confirm the results of blood cultures [13]. The protocol of the present study center prescribes meropenem for GNR bacteremia detected in a blood culture. GNR bacteremia occurs mostly in children with broad-spectrum antimicrobials, hospitalization in advanced healthcare settings (PICU, NICU), and the presence of central venous catheters [14,15]. As these patients are critically ill at the onset of GNR bacteremia, broad-spectrum antimicrobial therapy with meropenem is empirically used pending culture results. Antimicrobial susceptibility reports in the present study indicated that ESBL-producing Enterobacteriaceae were the leading source of antimicrobial resistance in GNR and that 20% of *Escherichia coli* produced ESBL. The most common ESBL type in Japan is *CTX-M* [16]. The multiplex PCR in the present study was not used to detect the *CTX-M* gene; therefore, ESBL-producing GNR was unable to be ruled out, and the decision to avoid meropenem was unable to be made before conventional susceptibility test results became available. The latest BioFire blood culture multiplex PCR panel is able to detect the *CTX-M* gene, which is harbored by the majority of ESBL-producing pathogens. It may, therefore, be possible to avoid using meropenem in patients with GNR bacteremia without the *CTX-M* gene to improve the initial response to non-carbapenem therapy.

The present study has several limitations. First, the study was conducted at a tertiary pediatric hospital, where many of the patients have an underlying medical condition. Thus, the findings may not be generalizable to children without comorbidities. Replication in different settings (e.g., community hospitals, adult populations) or different geographical locations might yield varying results.

Second, the study was retrospective and observational; thus, selection bias may have been introduced. Moreover, because the study had no control group other than the patients with multiplex PCR use, the possibility cannot be ruled out that the improvement in the outcomes stemmed from a natural improvement in clinical practice over time, which would occur independently of the use of multiplex PCR.

Third, the present study used a FilmArrayⓇ blood culture panel. The use of another multiplex PCR may not yield the same results as this study. However, other multiplex PCRs have been reported to be as sensitive as FilmArrayⓇ [17-19], and these test kits can detect similar resistance genes and bacteria, so other test kits can be substituted.

## Conclusions

This study found that introducing multiplex PCR reduced the use of anti-MRSA antimicrobial agents but did not affect the use of carbapenems. Therefore, when the causative organisms are GPC, mainly *Staphylococcus aureus*, multiplex PCR is expected to significantly optimize antimicrobial use. However, for GNR, it may not significantly impact antimicrobial optimization because resistance genes in GNR are not adequately detected.

Introducing multiplex PCR for bacteremia may enable rapid identification of bacteria and some resistance mechanisms, aiding in antimicrobial optimization. If more GNR resistance genes can be identified in the future, the potential for optimizing antimicrobial use will further increase.
